# Endothelial cell senescence exacerbates pulmonary hypertension by inducing juxtacrine Notch signaling in smooth muscle cells

**DOI:** 10.1016/j.isci.2023.106662

**Published:** 2023-04-11

**Authors:** Risa Ramadhiani, Koji Ikeda, Kazuya Miyagawa, Gusty Rizky Tough Ryanto, Naoki Tamada, Yoko Suzuki, Yuhei Kirita, Satoaki Matoba, Ken-ichi Hirata, Noriaki Emoto

**Affiliations:** 1 Laboratory of Clinical Pharmaceutical Science, Kobe Pharmaceutical University, 4-19-1 Motoyamakitamachi, Higashinada, Kobe 658-8558, Japan; 2 Division of Cardiovascular Medicine, Department of Internal Medicine, Kobe University Graduate School of Medicine, 7-5-1 Kusunoki, Chuo, Kobe 6500017, Japan; 3 Department of Epidemiology for Longevity and Regional Health, Kyoto Prefectural University of Medicine, 465 Kajii, Kawaramachi-Hirokoji, Kamigyou, Kyoto 6028566, Japan; 4 Department of Cardiology and Nephrology, Kyoto Prefectural University of Medicine, 465 Kajii, Kawaramachi-Hirokoji, Kamigyou, Kyoto 6028566, Japan

**Keywords:** Biological sciences, Molecular biology, Cell biology

## Abstract

Pulmonary arterial hypertension (PAH) is a fatal disease characterized by a progressive increase in pulmonary artery pressure caused by pathological pulmonary artery remodeling. Here, we demonstrate that endothelial cell (EC) senescence plays a negative role in pulmonary hypertension via juxtacrine interaction with smooth muscle cells (SMCs). By using EC-specific progeroid mice, we discovered that EC progeria deteriorated vascular remodeling in the lungs, and exacerbated pulmonary hypertension in mice. Mechanistically, senescent ECs overexpressed Notch ligands, which resulted in increased Notch signaling and activated proliferation and migration capacities in neighboring SMCs. Pharmacological inhibition of Notch signaling reduced the effects of senescent ECs on SMCs functions *in vitro*, and improved the worsened pulmonary hypertension in EC-specific progeroid mice *in vivo*. Our findings show that EC senescence is a critical disease-modifying factor in PAH and that EC-mediated Notch signaling is a pharmacotherapeutic target for the treatment of PAH, particularly in the elderly.

## Highlights

• EC-specific progeroid mice show exacerbated pulmonary hypertension
• Senescent ECs overexpress Notch ligands
• Thereby, senescent ECs activate Notch signaling in SMCs in a juxtacrine manner
• Notch inhibition improves the worsened PH in EC-specific progeroid mice

## Graphical abstract

**Figure:**
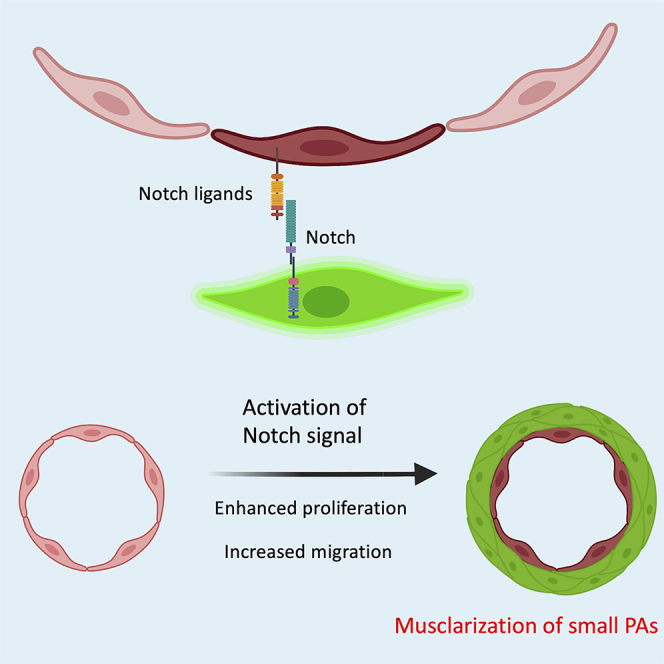

## Introduction

Pulmonary arterial hypertension (PAH) is a deadly lung disease characterized by progressive vasculopathy of small pulmonary arteries, resulting in elevated pulmonary arterial pressure and right heart failure.^1^^,^^2^ The recent development of new drugs for PAH greatly improved patients’ quality of life, hemodynamic parameters, and clinical outcomes. However, the long-term prognosis is still unsatisfactory, with a 5-year survival rate of ∼65%.^3^^,^^4^^,^^5^^,^^6^ Postmortem examination of the lungs of PAH patients whose symptoms were well-controlled on prostacyclin analog revealed extensive plexogenic arteriopathy, a hallmark of PAH.^7^ These findings suggest that disease progression is unavoidable despite currently therapies, which are primarily vasodilators, resulting in poor long-term survival in PAH. Thereby, new therapies targeting the pathological vascular remodeling to improve the vasculopathy are urgently needed.

The average age of idiopathic PAH patients is increasing; e.g. the mean age of PAH patients enrolled between 1981 and 1985 in the US registry was 36 years at the time of diagnosis, whereas the mean age of patients at diagnosis was 65 years in Germany in 2014 and was 69 years in a recent Swedish registry. Elderly PAH patients have a higher mortality rate and a lower response to clinical therapies than younger patients.^8^ Frequent comorbidities greatly contribute to the high mortality rate of older PAH patients, whereas aging may uniquely deteriorate the pathophysiology of PAH via an unknown mechanism.

Several animal studies have shown that hemodynamic unloading reverses the pathological occlusive lesion in pulmonary hypertension.^9^^,^^10^^,^^11^ These studies are consistent with the clinical findings of the regression of vasculopathy lesions after unilateral lung transplantation in the non-transplanted lung.^12^^,^^13^ In the case of PAH associated with congenital heart disease (PAH-CHD), hemodynamic unloading via shunt closure restores the pulmonary artery pressure and reverses the occlusive arteriopathy lesions in a short time. However, hemodynamic correction failed to maintain the lesion regression, and irreversible phenotypes resembling neointimal and plexiform lesions of PAH occurred after a certain period. Cellular senescence has recently been identified as the cause of reversibility loss in PAH associated with hemodynamic abnormalities.^14^^,^^15^^,^^16^^,^^17^ Furthermore, a crucial role of EC senescence caused by impaired iron-sulfur biogenesis because of frataxin deficiency has been implicated in the pathogenesis of pulmonary hypertension (PH).^18^

Cellular senescence was initially defined as a stable cell-cycle arrest caused by the limited proliferation capacity of cells, i.e., replicative senescence. The emerging evidence indicates another type of senescence, namely premature senescence, which is a stress response triggered by a variety of stimuli.^19^ Both types of senescent cells have been identified as a driver of age-related disease because of their ability to alter tissue homeostasis and promote secondary senescence via the senescence-associated secretory phenotype (SASP).^20^^,^^21^^,^^22^ SASP is crucial in the connection between vascular senescence and PH.^23^ Recent research has revealed that SASP is not the only mediator of non-autonomous functionality of cellular senescence, and that Notch signaling is also involved in the secondary senescence.^24^^,^^25^ Here, we investigated the potential role of EC senescence in the pathogenesis of PAH and discovered that it has a negative effect on the progression of PH via activation of juxtacrine Notch signaling in vascular smooth muscle cells (SMCs).

## Results

### EC-specific progeroid mice exhibit the exacerbated pulmonary hypertension

**Figure 1:**
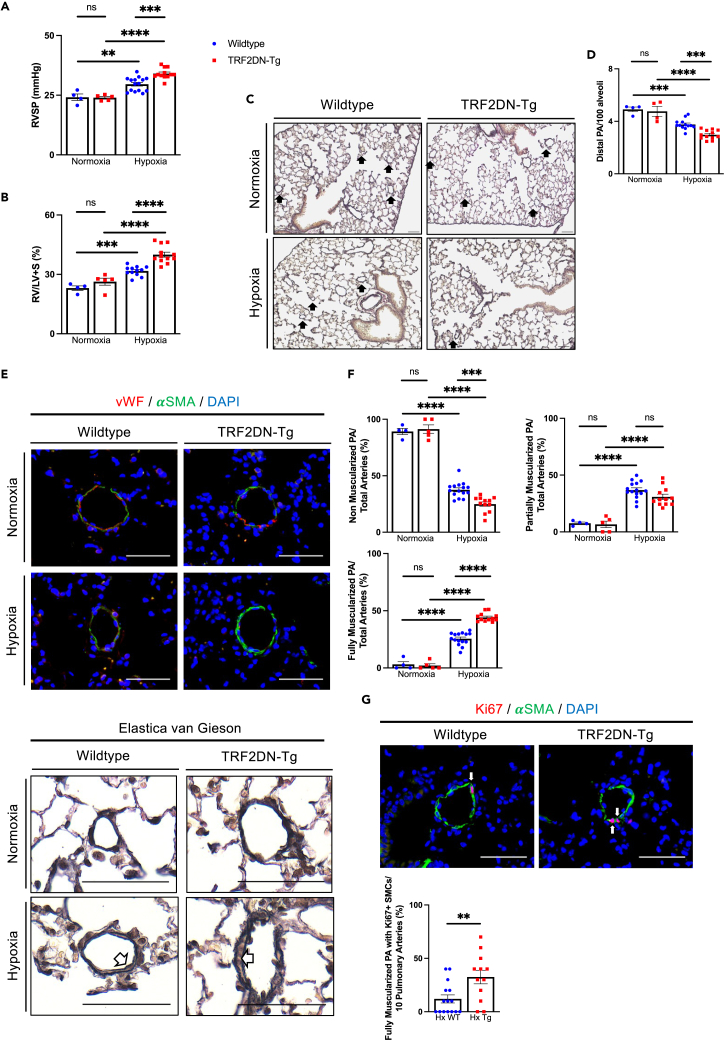
EC-specific progeria exacerbates pulmonary hypertension in mice (A and B) Right ventricular systolic pressure (RVSP) (A) and Ratio of right ventricle compared to left ventricle + septum (B) in WT and TRF2DN-Tg mice exposed to either normoxia or hypoxia for 3 weeks. (C) Representative images of the lung sections stained with Elastica van Gieson. Distal pulmonary arteries (PAs) are indicated by arrows. The Lungs dissected from WT and Tg mice exposed to either normoxia or hypoxia were analyzed. (D) Quantitative analysis for the number of distal PAs in the lungs of WT and Tg mice exposed to either normoxia or hypoxia. (E) Immunohistochemistry for von Willebrand factor (vWF) and α-smooth muscle actin (αSMA) in the lungs of WT and Tg mice exposed to either normoxia or hypoxia (upper images). Images of the lung sectioned stained with Elastica van Gieson were also shown (lower images). Distal PAs with medial thickening were indicated by arrows. (F) Quantification of non-, partial-, and fully-muscularized distal PAs in the lungs of WT and Tg mice exposed to either normoxia or hypoxia. (G) Immunohistochemistry for Ki-67 and αSMA in the lungs of WT and Tg mice exposed to hypoxia. Arrows indicate the Ki-67-positive proliferating smooth muscle cells (SMCs). Quantitative analysis for Ki-67 positive SMCs among 10 fully muscularized PAs was shown. Data are presented as mean ± SEM. Two-tailed Student’s *t* test was used for the analysis of the differences between two groups. Two-way ANOVA with Tukey’s post hoc test was used for the analysis of the differences between groups more than three. The number of samples was: n = 4 for normoxic WT; n = 4–5 for normoxic Tg; n = 12–15 for hypoxic WT; n = 12 for hypoxic Tg. Scale bars: 50 μm.*∗*p*<0.05, ∗∗*p*<0.01, ∗∗∗*p*<0.001, ∗∗∗∗*p*<0.0001,* and *ns;* not significant. See also Figures S1–S4.

We recently created EC-specific progeroid mice that overexpress the dominant-negative form of telomeric repeat-binding factor 2 (TRF2DN) in ECs under the control of the Tie2 or vascular endothelial cadherin (VEcad) promoter.^26^^,^^27^ ECs are particularly senescent in these mice, and EC-progeria caused impaired metabolic health, deteriorated atherosclerosis, and gliovascular interface abnormality in the brain.^26^^,^^27^^,^^28^ Using these EC-specific progeroid mice, we assessed the role of EC senescence in PH. Under the normoxic conditions, there were no differences in pulmonary arterial pressure or systemic hemodynamics between WT and VEcad-TRF2DN-Tg mice (Figures 1A and S1). Furthermore, there was no discernible defect in the pulmonary vasculatures of VEcad-TRF2DN-Tg mice (Figure S2). After three weeks of exposure to hypoxia (10% O_2_), VEcad-TRF2DN-Tg mice had worsened PH, as evidenced by the higher right ventricular systolic pressure (RVSP) and increased right ventricular mass normalized by left ventricle + septum mass (Fulton’s index) (Figures 1A and 1B). Histological analysis of the lungs revealed a further reduction in distal pulmonary arteries (PAs) (Figures 1C, 1D, and S3A) and worsened medial thickening in small PAs (Figures 1E, 1F, S3B, and S4) in VEcad-TRF2DN-Tg mice compared to WT mice after chronic hypoxia exposure. Notably, the number of medial proliferating SMCs in small PAs was significantly higher in the lungs of VEcad-TRF2DN-Tg mice than in WT mice (Figure 1G). These findings strongly suggest that EC senescence, in conjunction with increased SMCs proliferation, plays a negative role in PH.

### Senescent ECs promote SMCs proliferation and migration in the presence of cell-cell contact

**Figure 2:**
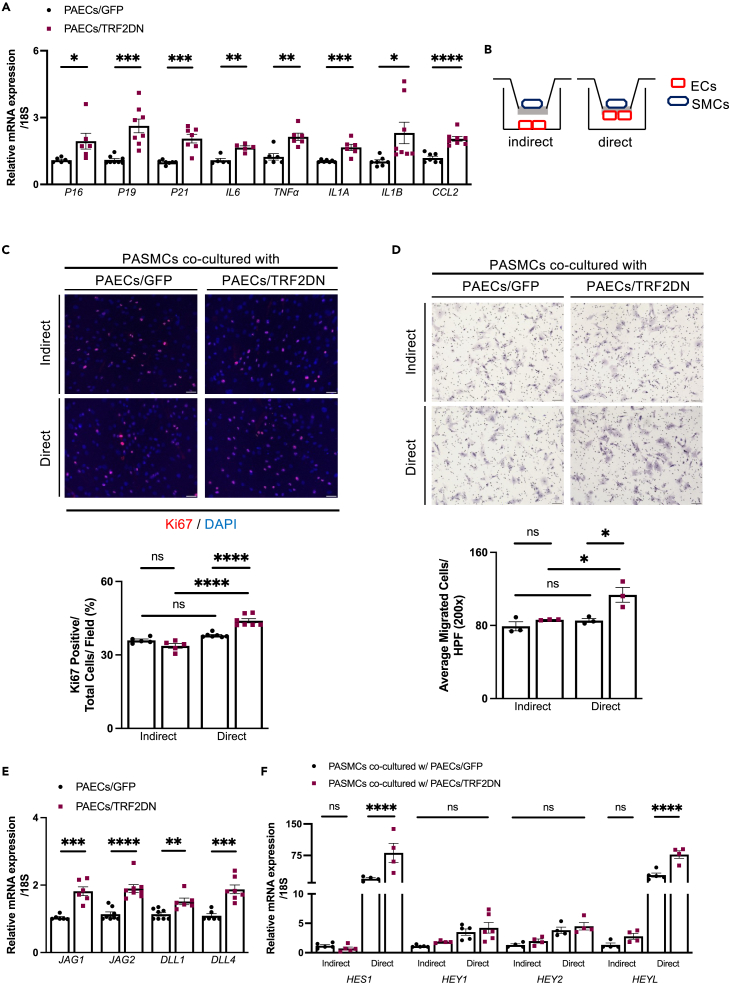
Senescent ECs enhance SMCs proliferation and migration through direct cell-cell contact (A) Real-time qPCR analysis for CDKIs and SASP factors in pulmonary artery ECs (PAECs) transfected with either GFP (control cells) or TRF2DN (premature senescent cells) (n = 5–8 each). (B) Schemes for co-culture experiments of PAECs and pulmonary artery SMCs (PASMCs). (C) Immunocytochemistry for Ki-67 in PASMCs directly (n = 7 each) or indirectly (n = 5 each) co-cultured with control or premature senescent PAECs. (D) Migration capacity was assessed by a modified Boyden chamber assay in PASMCs directly or indirectly co-cultured with control or premature senescent PAECs (n = 3 each). (E) Real-time qPCR analysis for Notch ligands in PAECs transfected with either GFP (control cells) or TRF2DN (premature senescent cells) (n= 6–8 each). (F) Real-time qPCR for Notch target genes in PASMCs directly or indirectly co-cultured with control or premature senescent PAECs (n = 4–5 each). Data are presented as mean ± SEM. Two-tailed Student’s *t* test was used for the analysis of the differences between two groups. Two-way ANOVA with Tukey’s post hoc test was used for the analysis of the differences between groups more than three. *∗*p*<0.05, ∗∗*p*<0.01, ∗∗∗*p*<0.001, ∗∗∗∗*p*<0.0001,* and *ns;* not significant. See also Figures S5–S7.

To investigate the molecular mechanisms underlying the exacerbated PH in EC-specific progeroid mice, we prepared senescent ECs by overexpressing the TRF2DN *in vitro*. Cellular senescence was confirmed by increased CDK inhibitors and SASP factors expressions as compared to those in GFP-transfected control cells (Figure 2A). Because loss of frataxin is associated with EC senescence,^18^ we examined its expression in these premature senescent ECs and found no difference from that in control cells (Figure S5A). Also, frataxin expression in mouse lung ECs was comparable in WT and VEcad-TRF2DN-Tg mice (Figure S5B). Because medial thickening with increased proliferation of SMCs was observed in VEcad-TRF2DN-Tg mice exposed to hypoxia, we investigated the effect of senescent ECs on SMCs functions. ECs and SMCs were co-cultured in two ways; direct (with cell-cell contact) and indirect (without cell-cell contact) fashion (Figure 2B). Direct interaction with ECs has been shown to promote the contractile phenotype of SMCs,^29^ and we confirmed morphological changes into a spindle shape as well as increased differentiation markers in the pulmonary artery (PA) SMCs directly co-cultured with ECs (Figures S6A and S6B). Notably, PASMCs directly co-cultured with senescent PAECs demonstrated increased proliferation and migration capacity when compared to PASMCs directly co-cultured with control GFP-transfected ECs (Figures 2C and 2D). Notably, these effects were not observed in the indirect co-culture condition (Figures 2C and 2D). PASMCs apoptosis was not significantly affected by the co-culture with senescent ECs both in direct and indirect conditions (Figure S7A). These findings suggest that the cell-cell contact-mediated interaction with senescent ECs boosts the proliferation and migration capacities in SMCs.

### Senescent PAECs enhance PASMCs migration and proliferation through Notch-mediated juxtacrine signaling

Senescent cells have distinct non-cell-autonomous functions via the senescence-associated secretory phenotype (SASP), which acts in an autocrine or paracrine manner.^30^^,^^31^ Recently, Notch signaling has been discovered as a mediator of senescent cells in a cell-contact-dependent juxtacrine fashion.^24^^,^^25^ We, therefore, assessed the possible role of Notch signaling in the communication between senescent ECs and SMCs. Expression of Notch ligands such as Jagged-1 (*JAG1*), Jagged-2 (*JAG2*), Delta-like ligand 1 (*DLL1*), and Delta-like ligand 4 (*DLL4*) were found to be significantly higher in senescent ECs than in control ECs (Figure 2E). Global patterns of DNA hypomethylation have been observed in senescent cells, which is associated with the altered expression in many genes.^32^ Pharmacological inhibition of DNA methylation using 5-azacytidine (5-AZA) partially repealed the increased Notch ligands expression in senescent ECs (Figure S6C). These findings suggest that senescence-associated epigenetic modifications are, at least partially, involved in the dysregulated Notch ligands expression in senescent ECs.

**Figure 3:**
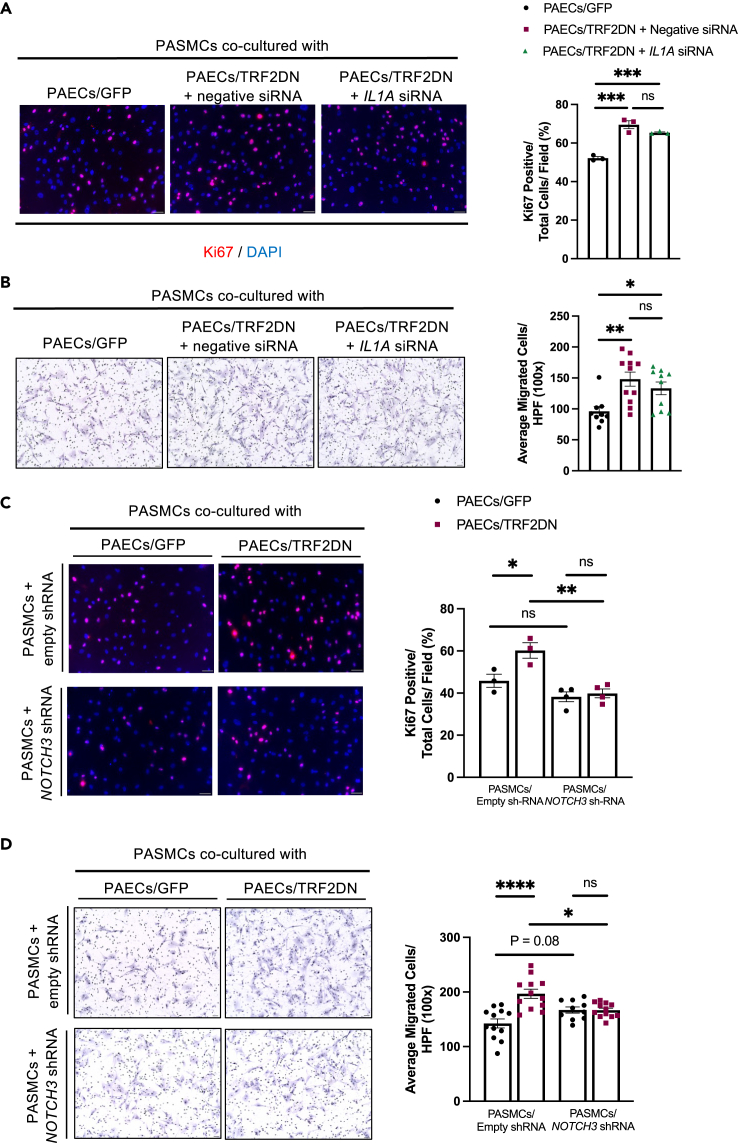
Notch signaling plays a crucial role in the pathological interaction between senescent ECs and SMCs (A) Immunocytochemistry for Ki-67 in PASMCs directly co-cultured with control (GFP) or premature senescent (TRF2DN) PAECs with or without IL-1a gene silencing (n = 3 each). (B) Migration capacity was assessed by a modified Boyden chamber assay in PASMCs directly co-cultured with control (GFP) or premature senescent (TRF2DN) PAECs with or without IL-1a gene silencing (n = 9–11 each). (C) Immunocytochemistry for Ki-67 in PASMCs directly co-cultured with control (GFP) or premature senescent (TRF2DN) PAECs (n = 3–4 each). PASMCs were infected with lentiviruses delivering either empty or Notch3 shRNA. (D) Migration capacity was assessed by a modified Boyden chamber assay in PASMC co-cultured with control (GFP) or premature senescent (TRF2DN) PAECs (n = 10–12 each). PASMCs were infected with lentiviruses delivering either empty or Notch3 shRNA. Data are presented as mean ± SEM. One-way ANOVA was used for the analysis of the differences between three groups. Two-way ANOVA with Tukey’s post hoc test was used for the analysis of the differences between groups more than three. *∗*p*<0.05, ∗∗*p*<0.01, ∗∗∗*p*<0.001, and ∗∗∗∗*p*<0.0001.* See also Figure S8.

Consistent with the increased Notch ligand expression, PASMCs directly co-cultured with senescent ECs had a higher transcription of Notch target genes such as *HES1* and *HEYL* than SMCs directly co-cultured with control ECs (Figure 2F). The γ-secretase inhibitor (DAPT), which inhibits Notch signaling, prevented the enhanced proliferation and migration in PASMCs directly co-cultured with senescent PAECs (Figures S7B and S7C). To investigate the role of SASP in the interaction between senescent ECs and SMCs in the presence of cell-cell contact, we conducted gene silencing of IL-1a, which orchestrates the SASP factors expression,^26^^,^^33^^,^^34^ in senescent ECs. Silencing of IL-1a reduced inflammatory cytokine expression in senescent ECs without affecting cellular senescence or Notch ligands expression (Figure S8A). Even after IL-1a silencing, senescent PAECs increased proliferation and migration in PASMCs that directly co-cultured with them (Figures 3A and 3B). Because Notch3 is critical in the pathogenesis of PH,^35^^,^^36^ we then performed gene silencing of Notch3 in PASMCs (Figure S8B). Silencing of Notch3 abolished the enhanced proliferation and migration in PASMCs directly co-cultured with senescent ECs (Figures 3C and 3D). These findings collectively indicate the critical role of Notch-mediated juxtacrine signaling in the enhanced capacities of proliferation and migration in PASMCs directly co-cultured with senescent PAECs.

**Figure 4:**
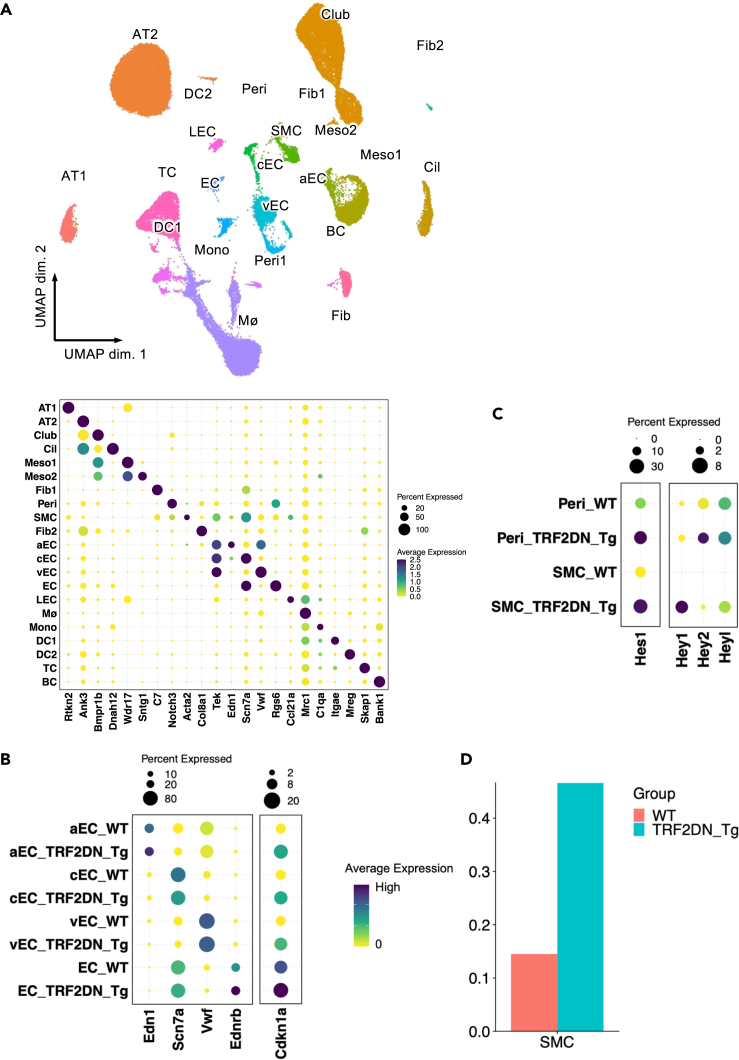
Single cell RNA-Seq analysis for the lungs (A) A total of 57,427 nuclei from two WT and two TRF2DN-Tg mice were projected by uniform manifold approximation and projection (UMAP) plot. AT1, alveolar type 1 epithelial cells; AT2, alveolar type 2 epithelial cells; Club, club cells; Meso, mesothelial cells; Fib, FIbroblasts; Peri, pericytes; SMC, smooth muscle cells; aEC, arterial endothelial cells; cEC, capillary endothelial cells; vEC, venous endothelial cell; EC, endothelial cells; LEC, lymphatic endothelial cells; Mø, macrophages; Mono, monocytes; DC = dendritic cells; TC, T cells; BC, B cells. Dot plot displaying gene expression patterns of cluster-enriched markers. (B) Dot plot displaying each EC cluster markers and Cdkn1a expression levels in the EC clusters. (C) Dot plot displaying gene expression patterns of Notch target genes in pericytes and SMCs. (D) Bar plot displaying the proportion of SMCs in each group. See also Figure S9.

Furthermore, we conducted single-cell nucleus RNA-Seq analysis using the lungs of WT and VEcad-TRF2DN-Tg mice exposed to chronic hypoxia (Figure 4A). We confirmed that CDK inhibitor expression in ECs was generally increased in arterial, capillary, and venous ECs of VEcad-TRF2DN-Tg mice when compared to WT mice (Figure 4B). Notably, Notch target gene expression was significantly increased in pericytes and/or SMCs of the Tg mice lungs when compared to WT mice (Figure 4C).

Moreover, the number of Acta2-positive SMCs increased in the lungs of VEcad-TRF2DN-Tg mice when compared to WT mice, which is consistent with the increased muscularization of small PAs in the lungs of the Tg mice (Figure 4D). These findings support the importance of the juxtacrine interaction between senescent ECs and pericytes/SMCs in the exacerbated pulmonary hypertension in EC-specific progeroid mice. In addition, we calculated the proportion of hyperproliferative ECs that actively contribute to human PAH.^18^ When compare to that reported in PAH patients (12.3%), the number of hyperproliferative ECs expressing Mki67 was relatively low in WT and VEcad-TRF2DN-Tg mice (Figure S9), which may explain the mild severity of PH in the hypoxia-induced PH model in mice.

### Inhibition of Notch signaling improves the PH phenotypes in EC-specific progeroid mice

Consistent with the *in vitro* findings, increased Notch ligands expression, such as *Jag1*, *Jag2*, *Dll1*, and *Dll4*, was discovered in pulmonary ECs isolated from VEcad-TRF2DN-Tg mice (Figure S10A). Furthermore, the expression of Notch target genes such as *Hes1*, *Hey1*, *Hey2*, and *HEYL*, in the lungs of VEcad-TRF2DN-Tg mice was significantly higher than that in WT mice (Figure S10B).

**Figure 5:**
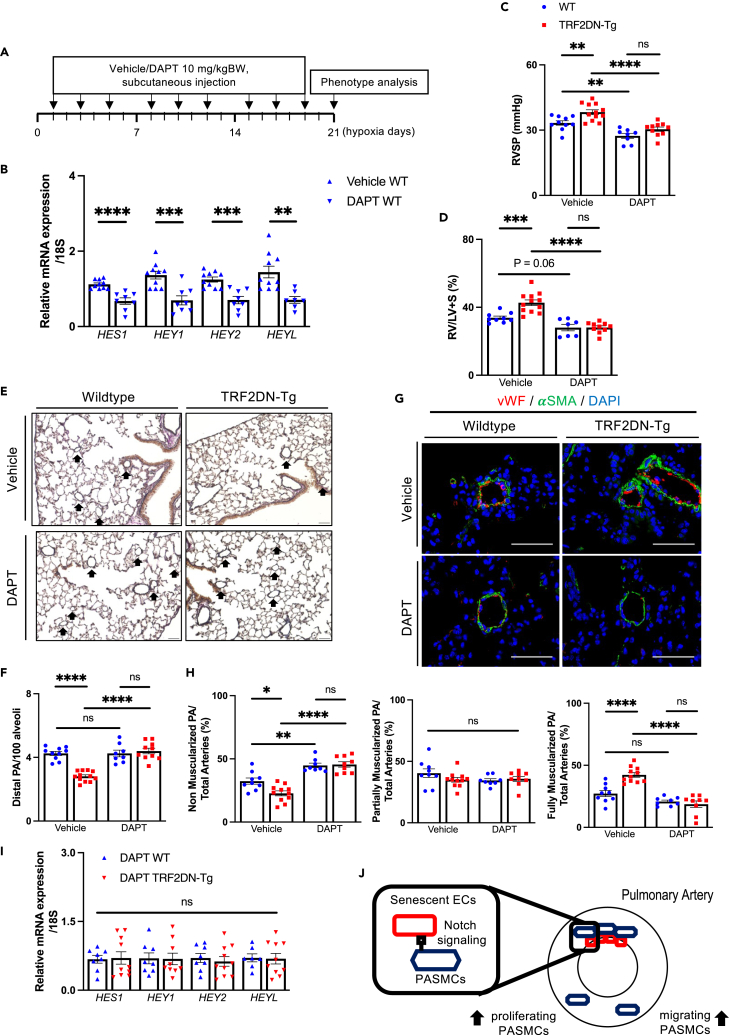
Inhibition of Notch signaling abolishes the exacerbated PH phenotype in EC-specific progeroid mice (A) Schematic diagram for mice experiments. (B) Real-time qPCR analysis for Notch target genes in whole lungs of WT mice treated with either vehicle or DAPT according to the experimental protocol shown in A. (C and D) RVSP (C) and Ratio of right ventricle compared to left ventricle + septum (D) in hypoxia-exposed WT and TRF2DN-Tg mice treated with either vehicle or DAPT. (E) Representative images of the lung sections stained with Elastica van gieson. The lungs dissected from hypoxia-exposed WT and TRF2DN-Tg mice treated with either vehicle or DAPT were analyzed. Arrows indicate distal PAs. (F) Quantitative analysis for the number of distal PAs in the lungs of hypoxia-exposed WT and TRF2DN-Tg mice treated with either vehicle or DAPT. (G) Immunohistochemistry for vWF and αSMA in the lungs of WT and TRF2DN-Tg mice treated with either vehicle or DAPT. (H) Quantitation for non-, partially, or fully-muscularized distal PAs in the lungs of hypoxia-exposed WT and TRF2DN-Tg mice treated with either vehicle or DAPT. (I) Real-time qPCR analysis for Notch target genes in whole lungs of hypoxia-exposed WT and TRF2DN-Tg mice treated with DAPT. (J) Schematic diagram for the mechanism underlying the detrimental role of senescent ECs in PH. Data are presented as mean ± SEM. Two-tailed Student’s *t* test was used for the analysis of the differences between two groups. Two-way ANOVA with Tukey’s post hoc test was used for the analysis of the differences between groups more than three. The number of samples was: n = 9–10 for vehicle-treated WT; n = 11–12 for vehicle-treated Tg; n = 7–8 for DAPT-treated WT; n = 9–10 for DAPT-treated Tg. Scale bars: 50 μm.*∗*p*<0.05, ∗∗*p*<0.01, ∗∗∗*p*<0.001, ∗∗∗∗*p*<0.0001,* and *ns;* not significant. See also Figure S10.

To elucidate a causative role of Notch signaling in the exacerbated PH phenotype in EC-specific progeroid mice, we administered either vehicle or DAPT three times a week during chronic hypoxia exposure, as shown in the experimental scheme (Figure 5A). Beforehand, pharmacological inhibition of Notch signaling was confirmed by the reduced Notch target genes expression in the lungs of WT mice treated with DAPT using this experimental protocol (Figure 5B). DAPT-treatment improved the PH phenotypes in both WT and VEcad-TRF2DN-Tg mice, as measured by a lower RVSP and reduced Fulton’s index ratio (Figures 5C and 5D). Notably, the exacerbated PH phenotypes in VEcad-TRF2DN-Tg mice vanished after DAPT treatment (Figures 5C and 5D). Histological examination of the lungs also revealed that DAPT treatment improved the worsened pulmonary artery remodeling in VEcad-TRF2DN-Tg mice (Figures 5E–5H). We confirmed that Notch target gene expression in the lungs was comparable in WT and VEcad-TRF2DN-Tg after treatment with DAPT (Figure 5I). These findings collectively suggest that senescent ECs worsen PH by increasing SMCs proliferation and migration capacities via enhanced Notch-mediated juxtacrine signaling (Figure 5J).

## Discussion

In this study, we discovered that senescent ECs play a negative role in the pathogenesis of PAH by interacting with PASMCs via Notch-mediated juxtacrine signaling. EC senescence is thought to play a causative role in age-related metabolic^26^^,^^37^ and cardiovascular disease.^27^^,^^30^^,^^38^^,^^39^^,^^40^^,^^41^ Furthermore, the potential role of cellular senescence has been investigated in non-age-related diseases such as type-1 diabetes^42^ and PAH associated with congenital heart disease.^16^ Senescence-associated secretory phenotype (SASP) has been identified as a non-cell-autonomous activity that can harm neighboring cells.^17^^,^^43^^,^^44^ Recently, Notch-mediated signaling has also been identified as a non-cell-autonomous function of cellular senescence in addition to the SASP.^24^^,^^25^^,^^45^

Senescent cells have massive transcriptional dysregulation as a result of epigenetic modifications such as global DNA hypomethylation, which may promote specific transcriptional programs.^46^ Earlier research found that various stimuli either transiently or persistently activate Notch signaling during cellular senescence process.^25^^,^^45^ In this study, we discovered that cellular senescence increased the expression of Notch ligands in ECs. Treatment with DNA methylation inhibitor, 5-AZA, increased *JAG1*, and *JAG2* expression, particularly in young control ECs, and thus abolished the difference in their expressions between young and senescent ECs. Therefore, senescence-associated DNA hypomethylation may be involved in the dysregulated Notch ligands expression in senescent ECs. However, 5-AZA treatment reduced *DLL4* expression in both young and senescent ECs, whereas the difference between the two disappeared. Therefore, increased Notch ligand expression in senescent ECs cannot be explained solely by DNA methylation status in these gene alleles, and more research is needed to understand the mechanisms underlying the senescence-associated Notch signaling alteration in pulmonary vasculatures.

One of the histopathological features of PAH-associated vascular remodeling is a remarkable medial thickening of distal pulmonary arteries, which are normally non-muscularized. Pathological medial thickening is caused by pre-existing SMCs that go through a process of dedifferentiation, distal migration, proliferation, and re-differentiation process.^47^ These phenotypic plasticities of SMCs are closely regulated by Notch3 signaling.^48^ Furthermore, Notch3 and its target gene HES5 are abundantly expressed in lung SMCs of PAH patients, and their expression levels are related to disease severity.^35^ Furthermore, Notch3-positive SMCs have been identified as the source of occlusive neointimal lesions of PAH.^36^ Notably, pharmacological, and genetic inhibition of Notch3 signaling reduced medial thickening and improved PH in mice.^35^^,^^36^ Therefore, Notch3 expressed in SMCs may serve as a primary receptor for Notch ligands expressed in senescent ECs. Indeed, gene silencing of Notch3 in PASMCs abolished the enhanced proliferation and migration capacities in cells directly co-cultured with senescent ECs in our experiments. However, more research is needed to determine the role of other Notch receptors in dysregulated Notch signaling in SMCs associated with EC senescence. Our findings indicated that SASP played a minor role in the pathological interaction between senescent ECs and SMCs in our experimental settings; however, these findings do not necessarily imply that SASP plays a minor role in the link between EC senescence and PAH. The contribution of SASP and the dysregulated Notch signaling to PH might vary depending on the disease status, and they may deteriorate PH in concert. Our current study highlights the importance of Notch signaling in the pathogenesis of PAH, and reveals a crucial role for ECs-SMCs juxtacrine interaction in the progression of PH, particularly in elderly patients.

Because cellular senescence plays an important role in aging and its associated diseases, eliminating senescent cells appears to be a promising strategy for delaying aging and/or alleviating age-related diseases. In fact, eliminating senescent cells has been shown to extend life span, and improve several age-related diseases such as atherosclerosis in mice.^49^^,^^50^^,^^51^^,^^52^ However, a recent study found that genetic and pharmacological removal of senescent cells exacerbates PH.^53^ The authors of this study elaborately demonstrated the detrimental effects of senescent cell clearance in pulmonary hemodynamics and PH by using various senolysis techniques and multiple PH animal models. Notably, ∼30% of pulmonary ECs express p16 even under normoxic conditions, and senescence features were detected primarily in microvascular ECs and SMCs in the lungs of iPAH patients and mice with PH. Their findings strongly suggest that cellular senescence in pulmonary vasculatures protects against PH, presumably by reducing their proliferation capacity. Because large number of pulmonary ECs showed senescence features, senolysis treatment reduced distal PAs and stimulated vessel remodeling in larger vessels without replacement of new cells. Although senolysis reduced SASP components in the lungs, the negative effects likely outweighed the benefits, and as a result, senolytic therapy worsens PH. These findings raise concerns about the use of senolytic interventions to treat age-related diseases. Especially when targeting PH with senescent ECs, functional correction, such as inhibiting the senescent EC-mediated juxtacrine Notch signaling, is preferable to eliminating senescent cells.

### Limitations of the study

In this study, we used EC-specific progeroid mice to investigate a role of EC senescence in the pathogenesis of PH. Although we previously showed the rationale for using these mice as a model of EC senescence *in vivo*, some differences in character may exist when compared to ECs of naturally aged mice.

## STAR★Methods

### Key resources table

**Table:** 

REAGENT or RESOURCE	SOURCE	IDENTIFIER
**Antibodies**		

Anti-von Willebrand Factor (Rabbit polyclonal)	Abcam	Ab9378; RRID: AB_307223
Anti-Actin, α-Smooth Muscle, FITC-linked (Mouse monoclonal)	Sigma-Aldrich	F3777; RRID: AB_476977
Anti-Ki67 (Rabbit monoclonal)	Histofine	418071; RRID: -
Alexa Fluor 594 donkey anti-rabbit IgG (Donkey polyclonal)	Life Technologies	A21207; RRID: AB_141637
CD146 (LSEC) Microbeads	Miltenyi Biotec	130-092-007; RRID: -
Anti-Cleaved Caspase 3 (Rabbit polyclonal)	Cell Signaling Technologies	9661S; RRID: AB_2341188
Anti-Total Cleaved Caspase 3 (Rabbit polyclonal)	Cell Signaling Technologies	9662S; RRID: AB_331439
Anti-GAPDH (Rabbit monoclonal)	Cell Signaling Technologies	2118S; RRID: AB_561053

**Chemicals, peptides, and recombinant proteins**		

DAPT (GSI-IX)	Selleckchem.com	S2215 Batch No.7; RRID: -
5-azacytidine (5-AZA)	Sigma-Aldrich	A2385; RRID: -

**Critical commercial assays**		

MACS lung dissociation kit, mouse	Miltenyi Biotec	130-095-927; RRID: -

**Deposited data**		

sc-RNAseq data	this paper	GSE228491

**Experimental models: Cell lines**		

Human PAECs	Promo Cell	C-12241; RRID: -
Human PASMCs	Promo Cell	C-12521; RRID: -

**Experimental models: Organisms/strains**		

VEcad-TRF2DN-Tg, strain background (C57Bl/6J)	https://doi.org/10.1038/s41467-020-14387-w	RRID: -

**Recombinant DNA**		

pLPC/TRF2-ΔB-ΔM (plasmid)	https://doi.org/10.1038/s41467-020-14387-w	RRID: Addgene_16069
pMSCVneo/GFP (plasmid)	https://doi.org/10.1038/s41467-020-14387-w	RRID: -
PVSVG (plasmid)	https://doi.org/10.1038/s41467-020-14387-w	RRID: -
SMARTvector lentiviral controls (empty vector)	Dharmacon	VSC11649
Notch3 shRNA (plasmid)	Santa Cruz	sc-37135-SH
*Silencer*™ Select Negative Control No. 2 siRNA	Invitrogen	4390846
siGENOME SMARTpool Human IL1A	Dharmacon	M-007952-01-0005

**Software and algorithms**		

Seurat v4	https://doi.org/10.1016/j.cell.2021.04.048	https://satijalab.org/seurat/
scds	https://doi.org/10.1093/bioinformatics/btz698	https://www.bioconductor.org/packages/release/bioc/html/scds.html
CellRanger	10x	https://support.10xgenomics.com/single-cell-gene-expression/software/downloads/latest
celda	https://doi.org/10.1093/nargab/lqac066	https://bioconductor.org/packages/release/bioc/html/celda.html
Graphpad Prism 9	Dotmatics	GPS-1627094-TLQG-360B1

### Resource availability

#### Lead contact

Further information and requests for resources and reagents should be directed to and will be fulfilled by the lead contact, Koji Ikeda (ikedak@koto.kpu-m.ac.jp).

#### Materials availability

All materials used in this study were shown in the key resources table.

### Experimental model and subject details

#### Animal model

All experimental protocols were approved by the Ethics Review Committee for Animal Experimentation of Kobe Pharmaceutical University. All researchers have complied with all relevant ethical regulations for animal testing and research, and animal experiments were performed in compliance with ARRIVE (Animal Research: Reporting of *In Vivo* Experiments) guidelines. Transgenic mice that overexpressed TRF2DN in EC (VEcad-TRF2DN-Tg) were generated (C57/BL6J background). The plasmid containing the TERF2-ΔB-ΔM was obtained from Addgene (plasmid #18008). The plasmid containing the VEcad-promoter was a gift from Dr. Mochizuki and Dr. Nakaoka (National Cardiovascular Research Center). The Tg mice were propagated as heterozygous Tg animals by breeding with WT C57/BL6J mice.

Mice were housed in designated cages of sufficient size (1-3 mice in one cage) in animal facility in which the temperature and humidity are regulated at ∼23 °C and ∼60%, respectively. Mice were maintained under a 12-h light/12-h dark cycle, and fed chow (normal chow diet containing 23.1% protein and 5.1% fat) with *ad libitum* access to water and food.

Male mice at 8–9 weeksold were regularly used for experiments. For chronic hypoxia exposure, mice were put in the chamber with non-recirculating gas mixture of 10% O_2_ and 90% N_2_ for 3 weeks. For DAPT experiments, vehicle (10% ethanol and 90% corn oil) or DAPT (10 mg/kg dissolved in 10% ethanol and 90% corn oil) was injected subcutaneously, 3 times a week, during chronic hypoxia exposure, in accordance with previous publications.^54^^,^^55^

#### Echocardiography

Transthoracic echocardiography was performed using a Siemens Acuson X300 connected to a VF13-5SP probe (Siemens) to visualize the heart. The heart rate, left ventricular end-diastolic diameter, left ventricular end-systolic diameter, aortic diameter, pulmonary artery accelerated time (PAAT) and aortic velocity-time integral were measured. Three measurements were taken for each parameter and averaged. The ejection fraction and CO were calculated using the respective formula.

#### Blood pressure measurements

The blood pressure was measured with a tail-cuff method using BP-98A-L (Softron) in a 37°C warmer without anesthesia. Five consecutive measurements were averaged. The results were presented in units of mmHg.

#### Right ventricular pressure measurements

Right heart catheterization was performed using 1.4 F Millar mikro-tip catheter transducer under anesthesia using ∼2% isoflurane inhalation. The catheter was inserted into right ventricle through right jugular vein. The right ventricular systolic pressure was calculated from the average of five pressure waves and presented in units of mmHg.

#### Fulton index measurements

The heart was dissected after 24–48 h fixation in 4% paraformaldehyde at 4°C. The right ventricle wall was separated from the left ventricle and septum and weighed separately. The data were presented as a ratio of the right ventricle to the left ventricle + septum.

### Method details

#### Histological analysis

The lung was inflated and fixed in 4% paraformaldehyde, followed by paraffin embedding. The sections were cut into 4 μm and stained with Elastica van Gieson, and the pulmonary arteries with diameter less than 50 μm was quantified as distal PAs. Images were captured using Keyence BZ-X800 microscopy (Keyence). In each mouse, 5 randomly selected images of terminal bronchioles (20x magnification) were taken and the number of distal PAs adjacent the terminal bronchiole were counted, followed by normalization with the number of alveoli, as shown in the Figure S3A. The data were presented as the number of distal PAs/100 alveoli.

Immunostaining was used to quantify the distal pulmonary artery muscularization. The lung sections were deparaffinized, followed by incubation in Antigen Unmasking Solution (Vector Laboratories) at 90°C for 10 min. The sections were blocked in 5% skim-milk in PBS with 0.2% Triton-Xprior to incubation with anti-von Willebrand factor (Abcam) and FITC-labeled anti-α-smooth muscle actin (Sigma-Aldrich) antibodies at 4°C overnight. Subsequently, the sections were incubated with fluorescence-labeled donkey anti-rabbit secondary antibodies (Invitrogen), followed by mounting with Vectashield mounting medium with DAPI (Vector Laboratories). Images were captured using Keyence BZ-X800 fluorescence microscopy (Keyence), and 5-6 randomly selected fields in lung periphery (20x magnification) were used for evaluation for each mouse. The distal PAs were determined as non-, partially-, and fully-muscularized by the existence of smooth muscle cells with <25%, 25–75%, and >75% of the circumference, respectively. Representative images for each muscularized PA are shown in Figure S3B. The data were presented as percent of non-, partially-, or fully muscularized PAs normalized with total number of vessels.

To assess the proliferation capacity of SMCs in distal pulmonary arteries, the immunostaining was performed as mentioned above using anti-Ki-67 (Nichirei) and anti-α-smooth muscle actin (Sigma-Aldrich) antibodies. The data were presented as percent of fully muscularized distal pulmonary arteries with Ki-67-positive SMCs.

#### Endothelial cell isolation

Isolation of mice lung endothelial cells was performed using gentle MACS Dissociator (Miltenyi Biotec Inc.), as indicated by the manufacturer. Briefly, 10–11 weeksold mice lungs were harvested, washed twice in PBS, cut into small pieces, and incubated in enzyme mix (Miltenyi Biotec Inc.). After 30 min incubation, homogenization of lungs were performed using gentleMACS Dissociators (#130-095-927, Miltenyi Biotec Inc.). The homogenate was filtered sequentially through 70 μm MACS Smart Strainer and 30 μm MACS Pre-Separation Filter. The dissociated cells were incubated with FcR Blocking reagent for mouse (#130-092-575, Miltenyi Biotec Inc.) for 10 minat 4°C, followed by incubation with CD146 (LSEC) MicroBeads (#130-092-007, Miltenyi Biotec Inc.) for 15 minat 4°C. Subsequently, cells were applied to LS column (#130-042-401, Miltenyi Biotec Inc.) in the magnetic field of MACS separator (#130-042-301, Miltenyi Biotec Inc.). After washing with PEB buffer for three times, isolated ECs were collected.

#### TRF2DN plasmid construction and retroviruses production

The plasmid containing the TRF2-ΔB-ΔM (deletion mutant lacking the N-terminal basic domain and C-terminal Myb domain) was obtained from Addgene (plasmid #18008).^26^ The TRF2DN/pMSCVneo and GFP/pMSCVneo construct were transfected into GP2-293 packaging cells using Lipofectamine 3000 (Thermo-Fisher) alongside with PVSVG viral envelope construct, followed by changing the medium in 24 h. After an additional 24 h incubation, fresh growth medium was given, and incubated for another 24 h. Subsequently, the culture medium containing retroviruses was collected and stored at -80°C after removal of cell debris by centrifugation.

#### Lentivirus production

Lentiviruses delivering empty shRNA was produced using SMARTvector Empty Vector Control with TurboGFP reporter (#VSC11649, Dharmacon), while lentiviruses delivering Notch3 shRNA was produced using Notch3 shRNA plasmid (#sc-37135-SH, Santa Cruz). These shRNA plasmids were transfected into 293T cells alongside with pMD2.G and psPAX2 plasmids using FuGENE-HD (Promega), followed by changing the medium in 24 h. After an additional 24 h incubation, fresh growth medium was given, and incubated for another 24 h. Subsequently, the culture medium containing lentiviruses was collected and stored at -80°C after removal of cell debris by centrifugation.

#### Quantitative real-time PCR

RNAs were extracted from the lungs or cells using RNAiso Plus (TAKARA), followed by purification using NucleoSpin RNA Clean-Up kit (Macherey-Nagel). The cDNA was synthesized from 0.5 and 1 μg of total RNA for cells and tissues, respectively, using PrimeScript RT Reagent Kit with gDNA eraser (TAKARA). Quantitative real-time PCR was performed using LightCycler96 (Roche Applied Science) with FastStart SYBR Green Master (Roche Applied Science). The mRNA expression levels of the target genes were normalized to 18S levels, and presented in arbitrary units. Nucleotide sequences of the primers are shown in Table S1.

#### Single nucleus RNA sequencing

Nuclei from two replicates of TRF2DN-Tg mice and WT mice lungs were isolated with Nuclei EZ Lysis buffer (Sigma) supplemented with protease inhibitor (Roche) and RNase inhibitor (Promega, Life Technologies). Samples were cut into < 2-mm pieces and homogenized using a Dounce homogenizer in 2 mL of ice-cold Nuclei EZ Lysis buffer. The homogenate was filtered through 100 μm and 40 μm cell strainers (pluriSelect) and then centrifuged at 500 × g for 5 minat 4°C. The pellet was resuspended in Nuclei Suspension Buffer (1x PBS, 1% bovine serum albumin, 0.2% RNase inhibitor), followed by filtration through a 5 μm cell strainer (pluriSelect). Nuclei were counted on hemocytometers (InCYTO C-chip) and partitioned into each droplet with a barcoded gel bead using the 10× Chromium instrument (10× Genomics). Single nuclei were lysed, and RNAs were reverse-transcribed into complementary DNA (cDNA) within each droplet. After breaking the emulsion, cDNAs were amplified and fragmented, followed by the addition of Illumina adapters using Single Cell 3′ Library & Gel Bead Kit. Samples were sequenced using Novaseq6000 (Illumina). Each sequencing data was processed by cellranger version 6.1.1 with “include-introns” option.

#### snRNA-seq data processing

Seurat v4 was used for downstream analyses including normalization, scaling and clustering of nuclei. Firstly, we analysed each replicates of samples separately and excluded low quality nuclei and multiplets identified with following strategy. After clustering with standard strategy according to Seurat-Guided Clustering Tutorial (https://satijalab.org/seurat/articles/pbmc3k_tutorial.html), we identified low quality nuclei with relatively high percentage of UMIs mapped to mitochondrial genes and relatively low number of genes, and multiplets with clusters with confused marker gene expression of multiple cell types and high doublet scores calculated with “cxds” function of the scds R package (https://www.bioconductor.org/packages/release/bioc/html/scds.html). Secondly, we used “decontX” function with default parameters of the celda R package to remove ambient RNA. Lastly, we corrected batch effect by using Seurat integration pipeline according to the instruction (https://satijalab.org/seurat/articles/integration_rpca.html).

#### Retroviral transfection in PAECs

Frozen stocks of the viruses were thawed immediately before use. PAECs at ∼70% confluence were infected with retroviruses using 1:1-2 mixture of retroviruses-containing medium and fresh growth medium. After 24 h incubation, fresh growth medium was given, followed by another 24 h incubation before use for experiments. The cells infected with retroviruses carrying GFP with more than 70% transfection efficacy were used as control cells.

#### Lentiviral transfection in PASMCs

Frozen stocks of the viruses were thawed immediately before use. PASMCs at ∼70% confluence were infected with lentiviruses using 1:1 mixture of lentiviruses-containing medium and fresh growth medium. After 24 h incubation, fresh growth medium was given, followed by another 24 h incubation before use for experiments. We regularly confirmed that control cells infected with lentiviruses carrying empty shRNA with GFP reporter showed more than 80% transfection efficacy.

#### Cell culture

Human pulmonary arterial endothelial cells (PAECs) and smooth muscle cells (PASMCs) were purchased from PromoCell. The donor of the PAECs was 23-year-old female caucasian, while the donor of the PASMCs was 30-year-old male caucasian. PAECs and PASMCs were cultured in HuMedia-EG2 (Kurabo) and Smooth Muscle Cells Growth Medium 2 (PromoCell), respectively at 37 °C under 5% CO_2_ levels in the CO_2_ incubator. All co-culture experiments were performed using 1 μm pore cell culture inserts (Falcon) coated with 1% gelatin (Wako). Indirect and direct co-culture was performed as previously described.^56^ Briefly, indirect co-culture was established by seeding GFP- or TRF2DN-transfected PAECs on the cell culture plate, and PASMCs were seeded on the cell culture insert. Direct co-culture was established by seeding GFP- or TRF2DN-transfected PAECs on the bottom of cell culture inserts followed by incubation for 2 h, and then PASMCs were seeded on the opposite side of the cell culture insert. In some experiments, premature senescent PAECs were transfected with 10 nM negative or IL-1a siRNA (Dharmacon) using lipofectamine RNAiMAX (Thermo-Fisher) before seeding for direct co-culture experiments. Also, PASMCs was infected with lentiviruses delivering either empty or Notch3 shRNA before seeding for direct co-culture experiments in some other experiments. After co-culturing for 48 h, the SMCs were assessed for their proliferation and migration capacities.

In some experiments, 10 μM DAPT dissolved in dimethyl sulfoxide (DMSO) or vehicle (DMSO) were added after overnight incubation of direct co-culture condition. After 48 h incubation in indirect or direct co-culture system, SMCs were assessed for proliferation, migration, and apoptosis capacities. Cells at passage 5 until 8 were used for all experiments.

In some experiments, PAECs were treated with 10 μM 5-azacytidine (5-AZA) dissolved in DMSO or vehicle (DMSO) for 72 h. Subsequently, cells were assessed for Notch ligands expression using Real-time qPCR.

#### Cell proliferation assessment by immunofluorescence staining

PAECs and PASMCs were plated in 12-well plate and 12-well cell culture insert, both indirect and direct co-culture conditions with seeding density of 5x10^4^ cells/well and 1.5x10^4^ cells/insert, respectively. PASMCs on cell culture inserts were fixed by 4% paraformaldehyde, followed by blocking with 5% skim milk for 1 h. The cells were then incubated with Ki-67 antibody (Nichirei) for overnight at 4°C. Subsequently, cells were incubated with fluorescence-labeled donkey anti-rabbit secondary antibodies (Invitrogen), followed by mounting with Vectashield mounting medium with DAPI (Vector Laboratories). The five randomly selected images (20x magnification) were taken using Keyence BZ-X800 fluorescence microscopy (Keyence) and used for quantification. The data were presented as a percentage of Ki-67-positive nuclei.

#### Modified Boyden chamber assay

PASMCs on cell culture inserts were trypsinized and seeded (20,000 cells per well) onto 8 μm pore inserts (Falcon). The number of migrated cells per well were assessed after 4 h, as previously described.^57^ After scraping off the remaining cells on the top of the insert using cotton swab, cells migrated onto the opposite side of the insert were fixed with methanol and then stained with hematoxylin. Subsequently, the insert was mounted onto a slide glass, followed by observation under the microscope. The number of cells were counted in randomly selected fields, and the mean number of cells were calculated for each insert.

#### Apoptosis assay

PAECs and PASMCs were plated in 6-well plate and 6-well cell culture insert, both indirect and direct co-culture conditions with seeding density of 10x10^4^ cells/well and 6x10^4^ cells/insert, respectively. To induce apoptosis, 500 nM hydrogen peroxide was added to PASMCs for 3 h. Cells were then trypsinized, followed by lyse in lysis buffer (10 mM Tris-HCl and 1% SDS), and boiled at 100°C for 5 min before centrifugation. Protein concentration was measured using the DC Protein Assay Kit (Bio-Rad).

Cells lysates were run on SDS-PAGE, and transferred onto a nitrocellulose membrane. Subsequently, the membranes were incubated with antibodies for cleaved caspase 3 (Cell Signaling Technology), total caspase 3 (Cell Signaling Technology), or GAPDH (Cell Signaling Technology). The chemiluminescence signals were detected by BioRad ChamiDoc XRS system (BioRad).

### Quantification and statistical analysis

#### Statistical analysis

All data were presented as the mean ± SEM. Statistical analysis was performed using Graphpad Prism 9. The difference between 2 groups was analysed using two-tailed unpaired student *t*-test, while differences between groups more than 3 were analyzed using one-way or two-way ANOVA with Tukey’s post hoc test, as indicated in the figure legend. *P*-value <0.05 was considered as statistically significant. The number of experiments and animals per group were indicated in the figure legends.

## Data Availability

This paper does not report original code. Any additional information required to reanalyze the data reported in this paper is available from the lead contact upon request. Raw and pre-processed data and metadata of the snRNAseq dataset have been deposited in NCBIs Gene Expression Omnibus and are available through GEO: GSE228491.
