# Supplementary material for: Endothelial cell senescence exacerbates pulmonary hypertension by inducing juxtacrine Notch signaling in smooth muscle cells
Source: iScience. 2023 Apr 11;26(5):106662. doi: 10.1016/j.isci.2023.106662 (PMC10182325; doi:10.1016/j.isci.2023.106662)
Supplement: Document S1. Figures S1–S10 and Table S1 [file mmc1.pdf]

iScience, Volume *26*

## Supplemental information

# Endothelial cell senescence exacerbates pulmonary hypertension by inducing juxtacrine Notch signaling in smooth muscle cells

**Risa Ramadhiani, Koji Ikeda, Kazuya Miyagawa, Gusty Rizky Tough Ryanto, Naoki Tamada, Yoko Suzuki, Yuhei Kirita, Satoaki Matoba, Ken-ichi Hirata, and Noriaki Emoto**

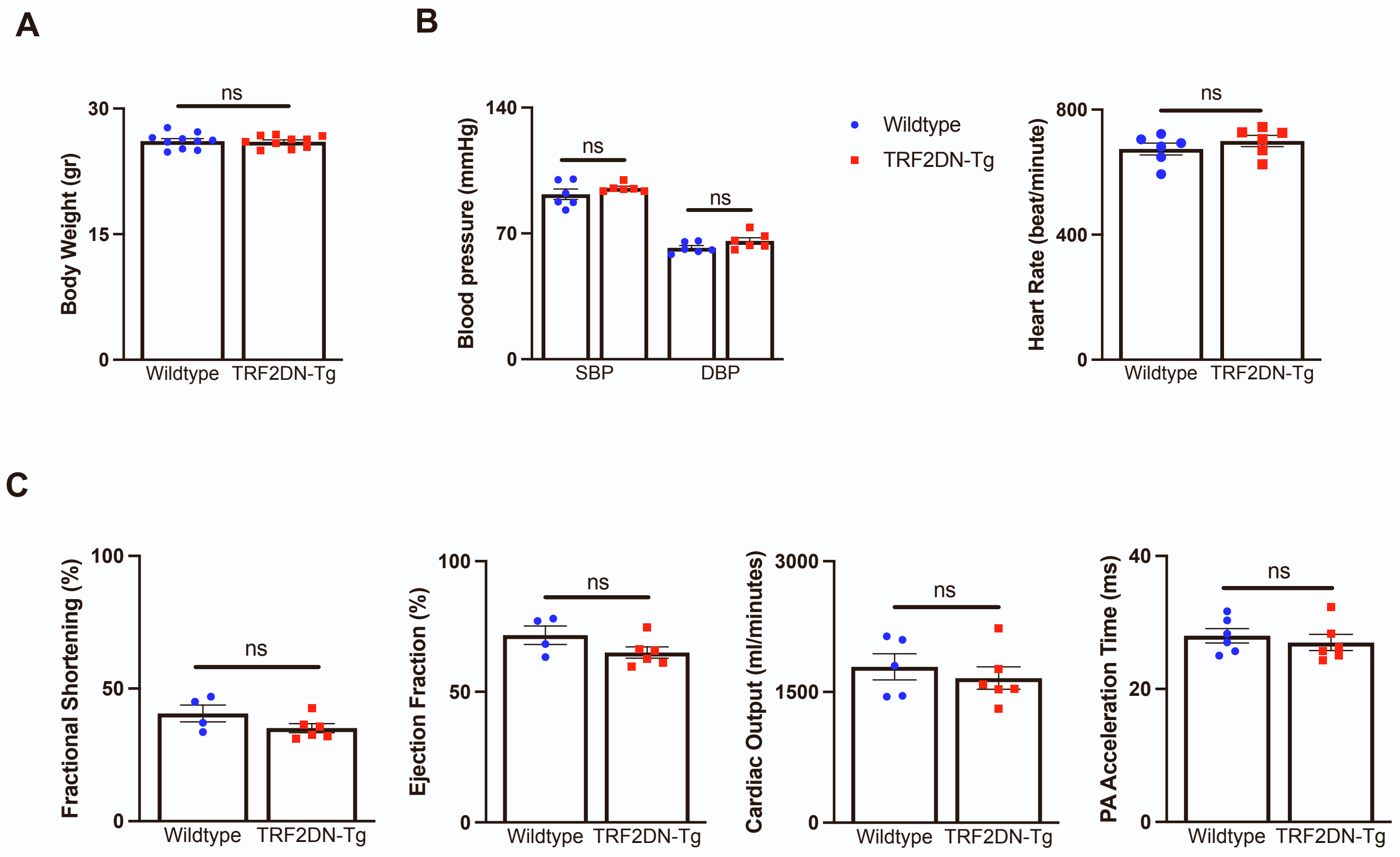


**Figure S1. Basic characteristics of WT and EC-specific progeroid mice, Related to Figure 1.** (A) Body weight in WT and TRF2DN-Tg mice at the time of hypoxia exposure (n = 10 each). (B) Blood pressure and heart rate in WT and TRF2DN-Tg mice (n = 6 each). (C) Echocardiography parameters in WT and TRF2DN-Tg mice (n = 4-6 for WT; n = 5-6 for Tg). Data are presented as mean ± SEM. Two-tailed student's *t*-test was used for the analysis of the differences between two groups. **P < 0.05, **P < 0.01, ***P < 0.001, ****P < 0.0001*, and *ns*; not significant.

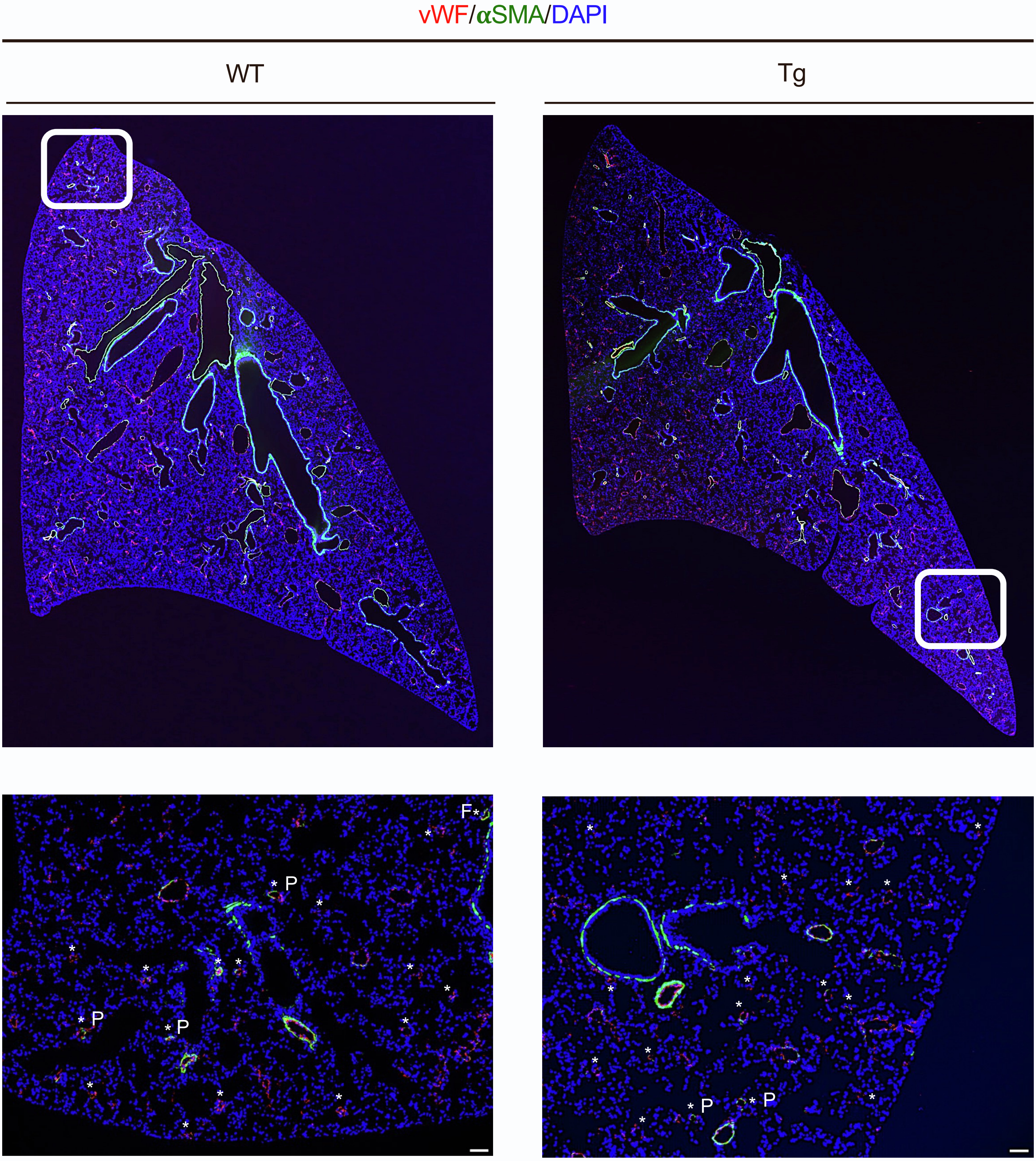


**Figure S2. Large panels of pulmonary vessel images in the lungs of mice in normoxic condition, Related to Figure 1.**

Immunohistochemistry for von Willebrand factor (vWF) (red) and αSMA (green) in the lungs of WT and Tg mice under normoxic condition. Nuclei were stained with DAPI (blue). Enlarged images for peripheral parts surrounded by white box were shown at the bottom. * represents non-muscularized small PAs (< 50 μm), *P represents partially-muscularized PAs, and *F represents fully-muscularized PAs. Scale bars: 50 μm.

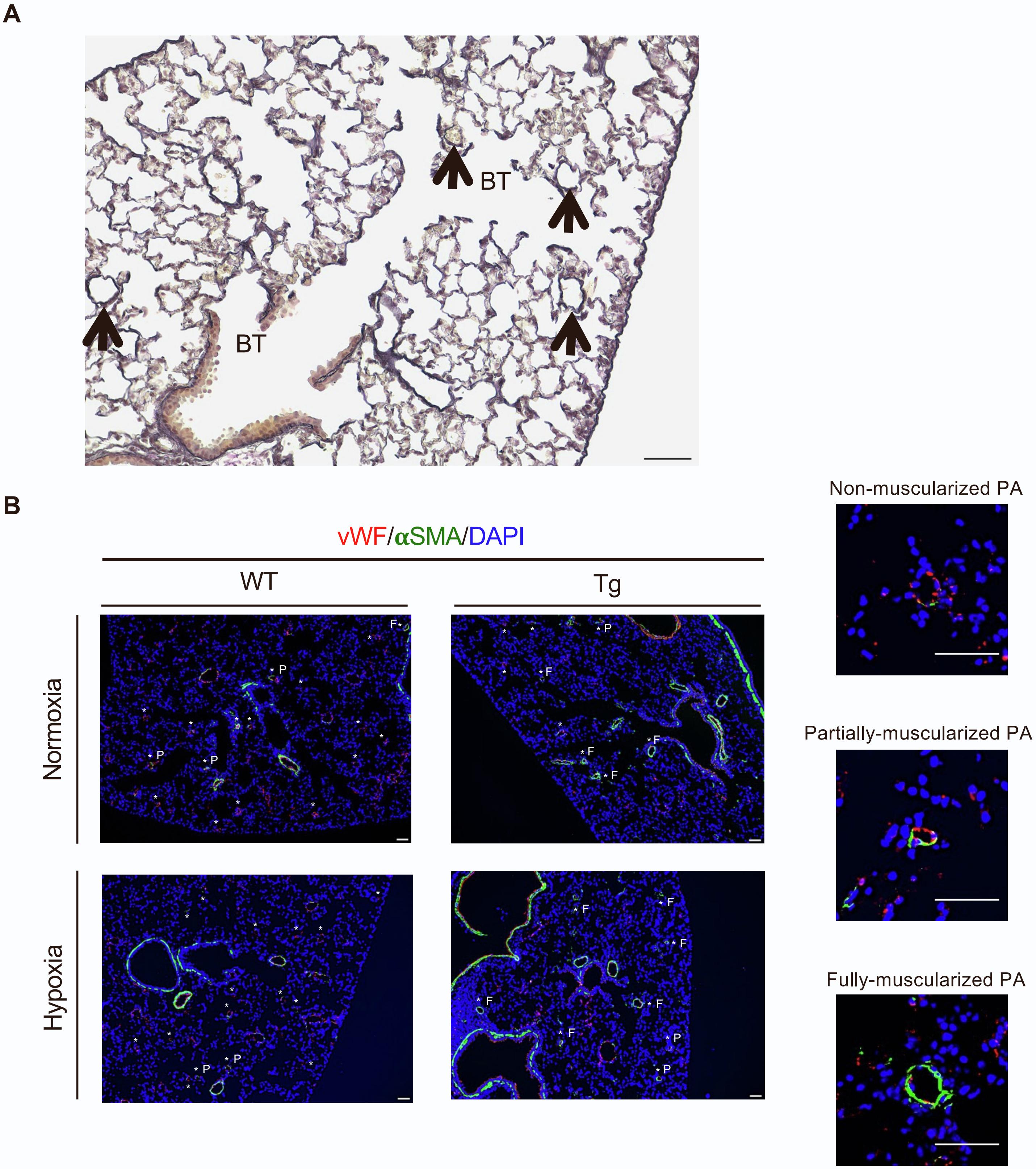


**Figure S3. The metrics for evaluation of the small PA muscularization, Related to Figure 1.** (A) Elastica van Gieson staining was used for quantification of small pulmonary arteries (PA). PAs with diameter of less than 50 μm were determined as small distal PAs (indicated by black arrows). The number of small distal PAs surrounding the bronchus terminalis (BT) was counted, and then normalized with the number of alveoli. (B) Representative images of immunostaining for vWF (red) and αSMA (green) in the lung sections. Nuclei were stained by DAPI (blue). The ratio of non-, partially-, and fully-muscularized small PA was shown by a percentage in all small PAs. For each mouse, 5-6 independent fields were analyzed, and mean value was calculated. The number of mice used in each group was described in each figure legend. * represents non-muscularized small PAs (< 50 μm), *P represents partially-muscularized PAs, and *F represents fully-muscularized PAs. Scale bars: 50 μm.

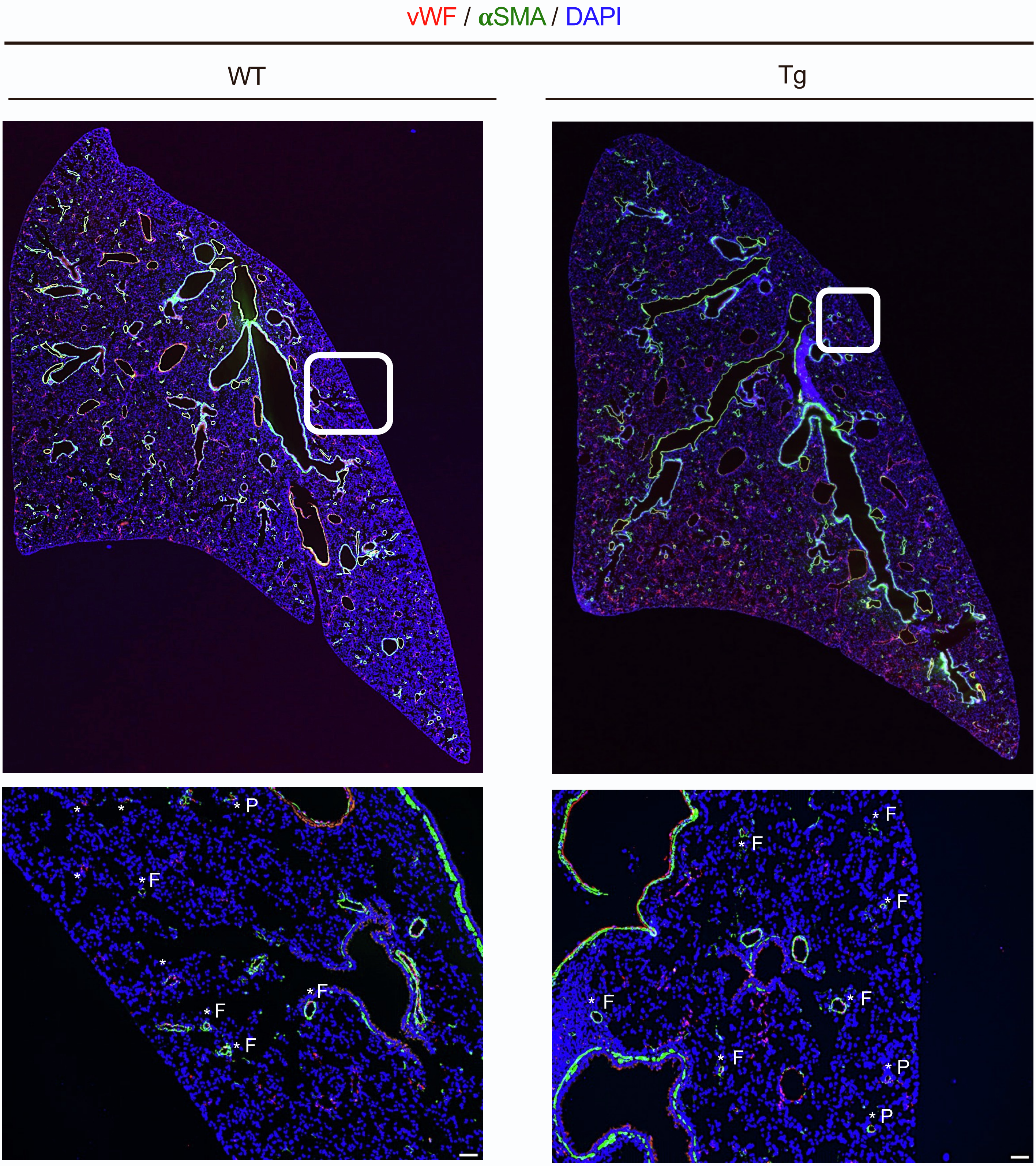


**Figure S4. Large panels of pulmonary vessel images in the lungs of mice in hypoxic condition, Related to Figure 1.**

Immunohistochemistry for von Willebrand factor (vWF) (red) and αSMA (green) in the lungs of WT and Tg mice under hypoxic condition. Nuclei were stained with DAPI (blue). Enlarged images for peripheral parts surrounded by white box were shown at the bottom. * represents non-muscularized small PAs (< 50 μm), *P represents partially-muscularized PAs, and *F represents fully-muscularized PAs. Scale bars: 50 μm.

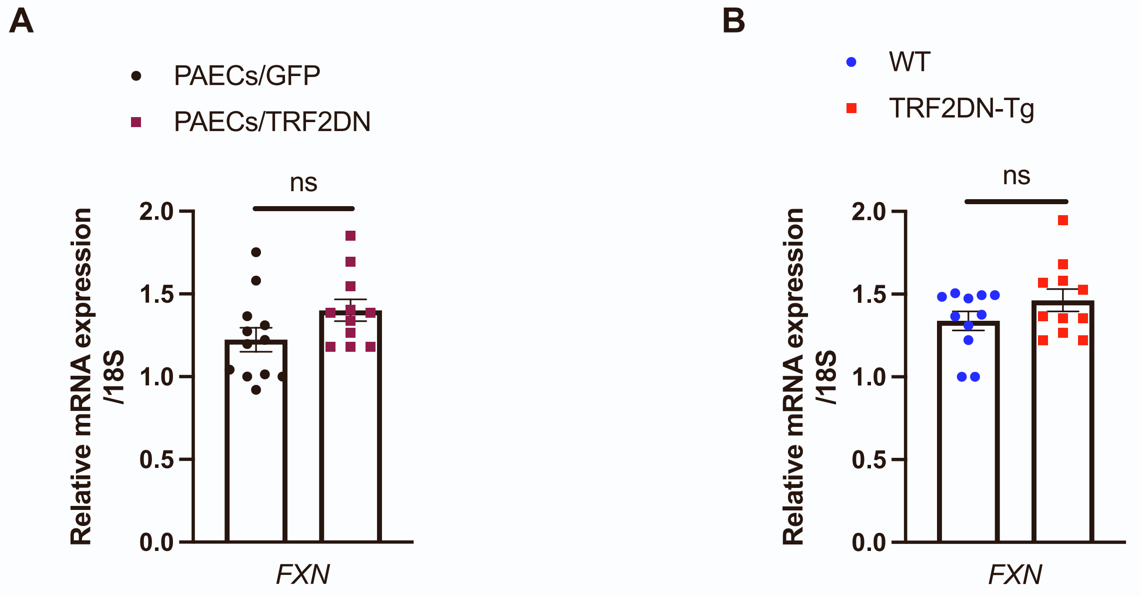


**Figure S5. FXN expression in ECs, Related to Figure 2.**
(A) Frataxin (FXN) mRNA expression was analyzed in PAECs transfected with either GFP or TRF2DN by qPCR (n = 11-12 each). (B) FXN mRNA expression was analyzed in ECs isolated from the lungs of WT or TRF2DN-Tg mice under normoxic condition (n = 11 each).

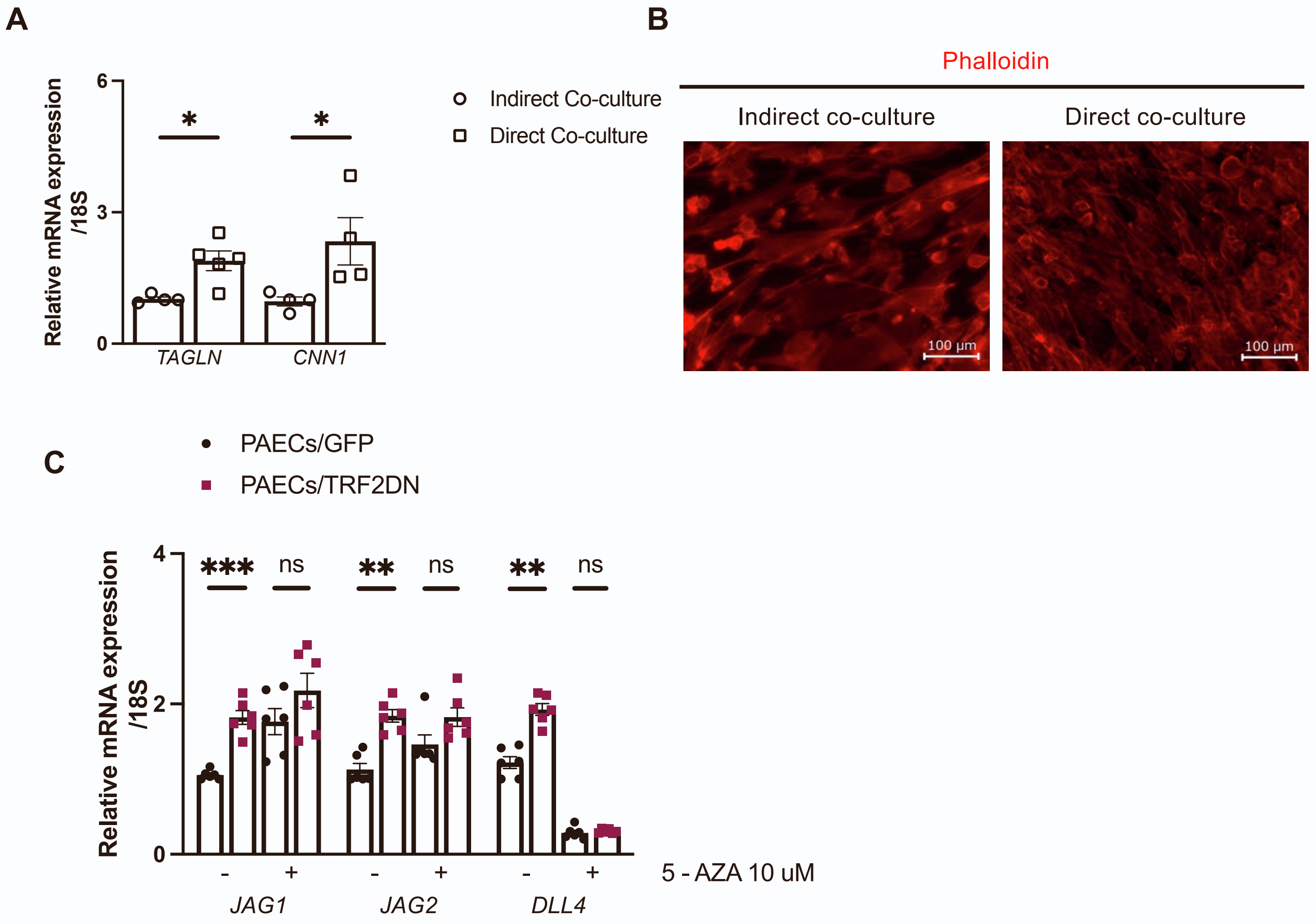


**Figure S6. Characteristic of PASMCs and PAECs in the co-culture condition, Related to Figure 2.** (A) Real-time qPCR analysis for differentiation markers in PASMCs directly (n = 4 each) or indirect (n = 4 each) co-cultured with PAECs. (B) Phalloidin staining in PASMCs directly or indirectly co-cultured with PAECs. (C) Real-time qPCR analysis for Notch ligands in PAECs transfected with either GFP (n = 6 each) or TRF2DN (n = 6 each). Cells were treated with with either vehicle or 10 μM 5-azacytidine (5-AZA). Data are presented as mean ± SEM. Two-tailed student's *t*-test was used for the analysis of the differences between two groups. Two-way ANOVA with Tukey's post hoc test was used for the analysis of the differences between groups more than three. **P < 0.05, **P < 0.01, ***P < 0.001, ****P < 0.0001*, and *ns*; not significant.

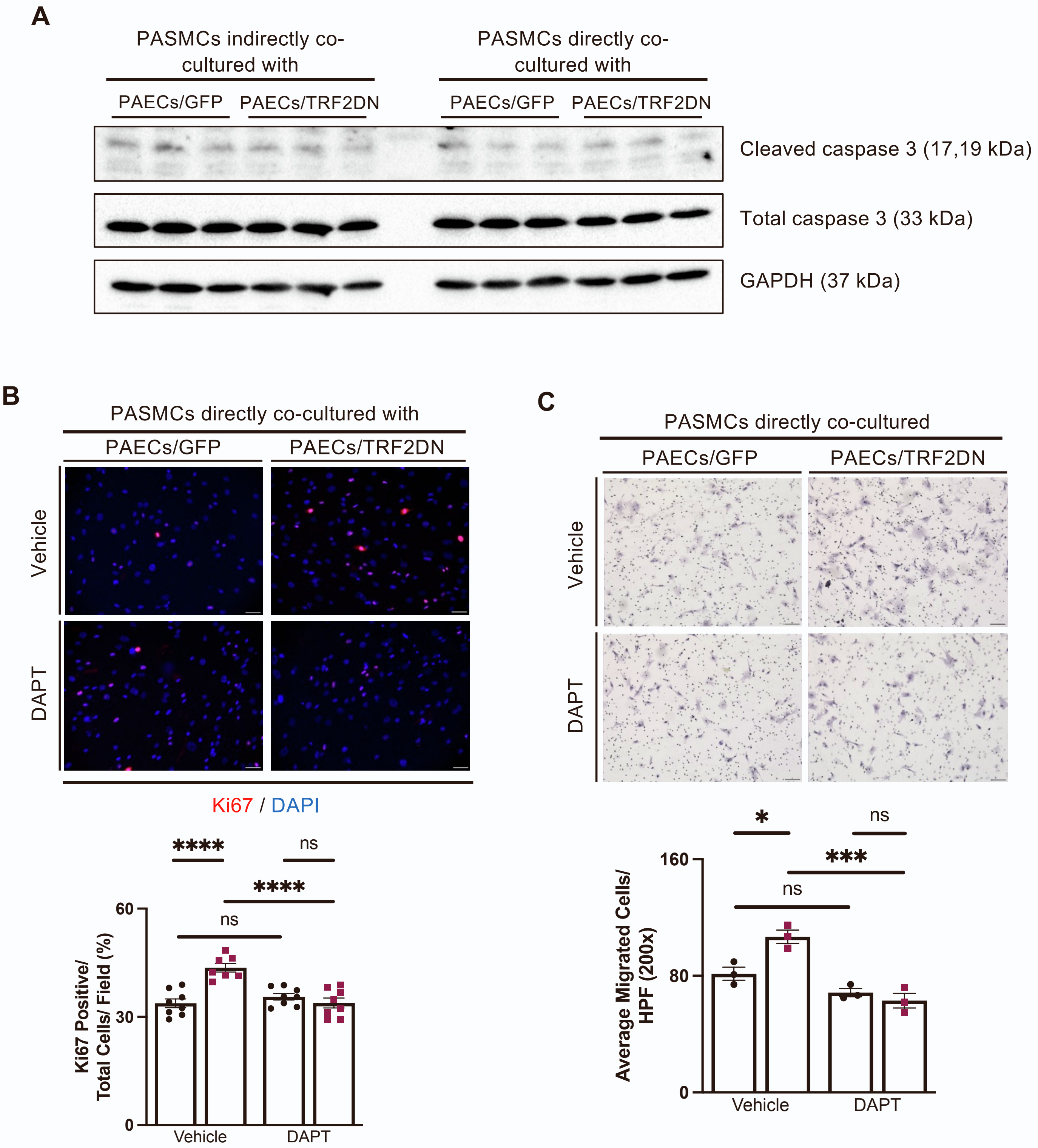


**Figure S7. Senescent ECs affect SMCs functions through Notch-mediated juxtacrine interaction, Related to Figure 2.**
(A) Immunoblotting for cleaved caspase-3, total caspase-3, and GAPDH in PASMCs directly or indirectly co-cultured with control or premature senescent PAECs (n = 3 each). Apoptosis was induced by incubating with 500 nM hydrogen peroxide for 3 h. (B) Immunocytochemistry for Ki-67 in PASMCs directly co-cultured with control or premature senescent PAECs. Cells were treated with either vehicle or 10 μM DAPT. Ki-67-positive PASMCs were quantified (n = 7-8 each). (C) Migration capacity was assessed by a modified Boyden chamber assay in PASMCs directly co-cultured with control or premature senescent PAECs. Cells were treated with either vehicle or 10 μM DAPT. Migrated PASMCs were quantified (n= 3 each). Data are presented as mean ± SEM. Two-way ANOVA with Tukey's post hoc test was used for the analysis of the differences between groups more than three. *$^*P < 0.05$, $^{**}P < 0.01$*, and *ns*; not significant.

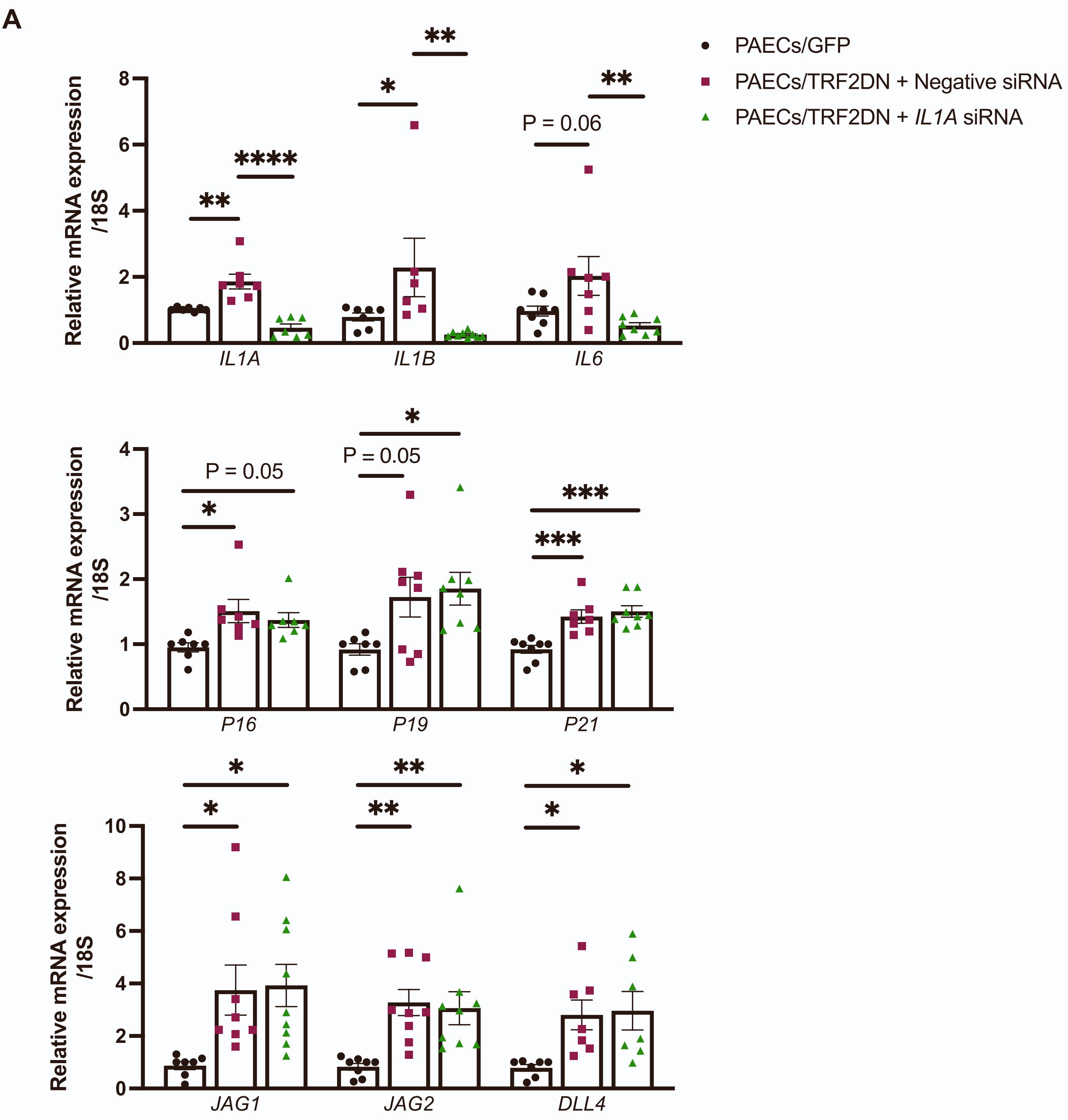


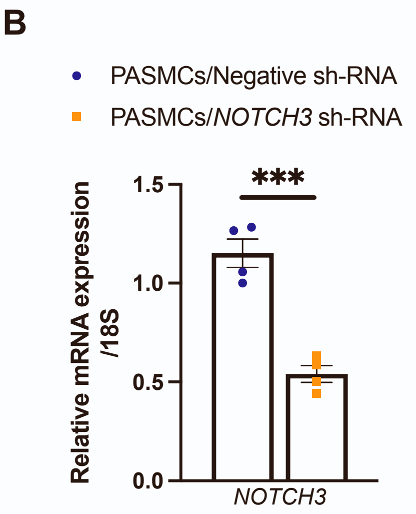


**Figure S8. Gene silencing of IL-1a in senescent ECs, and Notch3 in SMCs, Related to Figure 3.**

(A) Realtime qPCR analysis for CDKIs, SASP factors, and Notch ligands in PAECs transfected with GFP, TRF2DN + negative siRNA, and TRF2DN + IL-1a siRNA (n = 6-10 each). (B) Realtime qPCR analysis for Notch3 in PASMCs infected with lentiviruses delivering either empty and Notch3 shRNA (n = 4 each).

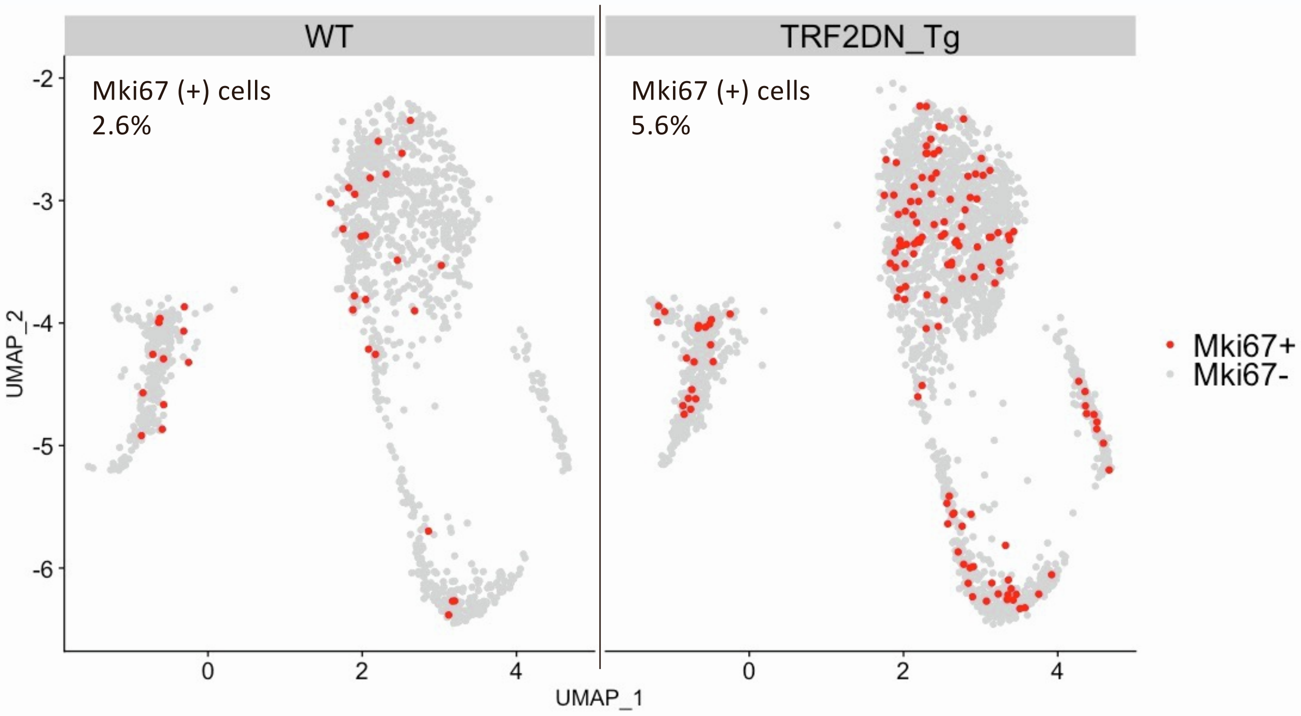


**Figure S9. UMAP plots of lung ECs, Related to Figure 4.**
From single-nucleus RNA-Seq of the lungs of WT and VEcad-TRF2DN-Tg mice exposed to chronic hypoxia, UMAP plots were generated of all ECs in each mouse. Mki67-positive ECs are marked as red plots.

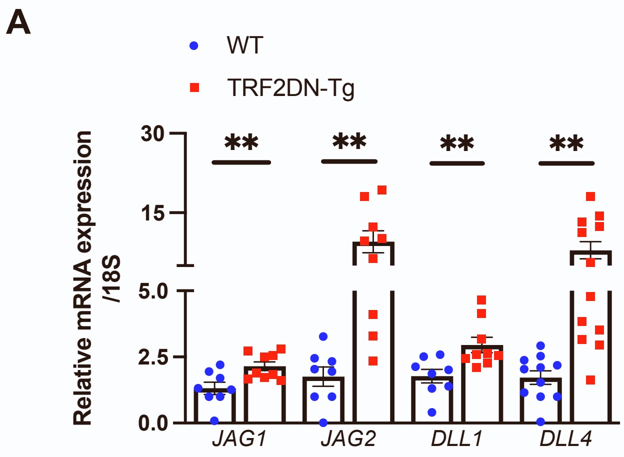


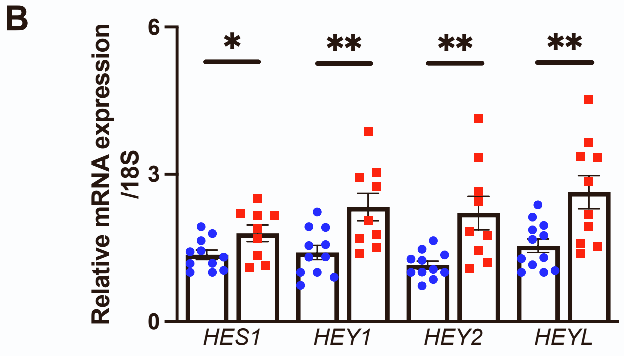


**Figure S10. Notch juxtacrine signaling is enhanced in the lungs of EC progeroid mice, Related to Figure 5.**

(A) Real-time qPCR analysis for Notch ligands in ECs isolated from the lungs of WT (n = 8-11) and TRF2DN-Tg (n = 9-10) mice exposed to chronic hypoxia. (B) Real-time qPCR analysis for Notch target genes in the lungs of WT (n = 11-12) and TRF2DN-Tg (n = 9-10) mice exposed to chronic hypoxia. Data are presented as mean ± SEM. Two-way ANOVA with Tukey's post hoc test was used for the analysis of the differences between groups more than three. **P < 0.05, and **P < 0.01.*

**Table S1. Primers for RT-qPCR**

| | |
|---|---|
| h18S rRNA_ F | GTAACCCGTTGAACCCCATT |
| h18S rRNA_ R | CCATCCAATCGGTAGTAGCG |
| hP16_F | CACCAGAGGCAGTAACCATGCCCGC |
| hP16_R | GTAGGACCTTCGGTGACTGATGATC |
| hP19_F | GGCAGTTCAAGAGGGTCACACTGCT |
| hP19_R | ACCATGTGGCCCTGCAGGATGTCCA |
| hP21_F | GGAAGACCATGTGGACCTGTCACTG |
| hP21_R | AGATCAGCCGGCGTTTGGAGTGGTA |
| hIL6_F | GAAGCTGCAGGCACAGAACCAGTGGC |
| hIL6_R | CTGACCAGAAGAAGGAATGCCCATT |
| hTNFα_F | GGCTGATTAGAGAGAGGTCC |
| hTNFα_R | CACTGAAAGCATGATCCGGG |
| hIL1A_F | GGTCACCAAATTCTACTTCCAGGAGGAC |
| hIL1A_R | GTGACCAGGTTGTTGTGACGCCTTC |
| hIL1B_F | AGCTGTACCCAGAGAGTCCTGTGCTGA |
| hIL1B_R | AGGAGAGAGCTGACTGTCCTGGCTGATG |
| hCCL2_F | GAAGAATCACCAGCAGCAAGTGTCCC |
| hCCL2_R | GCTTGTCCAGGTGGTCCATGGAATCC |
| hJAG1_F | TCGGGTCAGTTCGAGTTGGA |
| hJAG1_R | CGTTCACGTTCTGCATGGAC |
| hJAG2_F | GATACCA CCCCGAATGAGGAG |
| hJAG2_R | GGGTTGATCATGCCGGC |
| hDLL1_F | AACACCAACAAGAAGGCGGA |
| hDLL1_R | CCTCAGTTGCTATGACGCAC |
| hDLL4_F | GGCCAACTATGCTTGTGAATGTC |
| hDLL4_R | ACCTCGGTTCAGGCACTGTC |
| hHES1_F | CGGACATTCTGGAAATGACA |
| hHES1_R | TTGATCTGGGTCATGCAGTT |
| hHEY1_F | AGAGTGCGGACGAGAATGGAAACT |
| hHEY1_R | CGTCGGCGCTTCTCAATTATTCCT |
| hHEY2_F | TTGAAGATGCTTCAGGCAACAGGG |
| hHEY2_R | TCAGGTACCGCGCAACTTCTGTTA |
| hHEYL_F | ATGCAAGCCAGGAAGAAACGCAGA |
| hHEYL_R | AGCTTGGAAGAGCCCTGTTTCTCA |
| hTAGLN_F | CCTGGCTAGGGAAACCCACCCT |
| hTAGLN_R | TCTGGGGAAAGCTCCTTGGAAGT |
| hCNN1_F | AGCATGGCGAAGACGAAAGGAA |
| hCNN1_R | CCCATCTGCAGGCTGACATTGA |
| hFXN_F | CAGTGGACCTAAGCGTTATGACTGGA |
| hFXN_R | CAGACCTCAGCTGCATAATGAAGCTGG |
| hNOTCH3_F | GATGAGCTTGGGAAATCAGC |
| hNOTCH3_R | GATCTCACGGTTGGCAAAGT |

| Table S1. Primers for RT-qPCR | |
|---|---|
| m18S rRNA_ F | GTAACCCGTTGAACCCCATT |
| m18S rRNA_ R | CCATCCAATCGGTAGTAGCG |
| mJAG1_F | CAGTGCCTCTGTGAGACCAA |
| mJAG1_R | AGGGGTCAGAGAGACAAGCA |
| mJAG2_F | GGCAAAGAATGCAAAGAAGC |
| mJAG2_R | TGGCTGCCACAGTAGTTCAG |
| mDLL1_F | CCACGGTCAGGGATACACAC |
| mDLL1_R | GTGTTGGGGCGATCTTCTCT |
| mDLL4_F | ACCTTTGGCAATGTCTCCAC |
| mDLL4_R | TTGGATGATGATTTGGCTGA |
| mHES1_F | TCATGGAGAAGAGGCGAAGGGCA |
| mHES1_R | GAGCGCGGCGGTCATCTGC |
| mHEY1_F | GAGAAGCGCCGACGAGACCG |
| mHEY1_R | GGCGTGCGCGTCAAAATAACCTTT |
| mHEY2_F | TGCGTTCCGCTAGGCGACAG |
| mHEY2_R | TGAGCTTGTAGCGTGCCCAGG |
| mHEYL_F | CAGCCCTTCGCAGATGCAA |
| mHEYL_R | CCAATCGTCGCAATTCAGAAAG |
| mFXN_F | TTGGGGACATTGGACAACCC |
| mFXN_R | CTTCCCGGTCCAGTCATAGC |
